# Dissociative features of fibromyalgia syndrome

**DOI:** 10.17712/nsj.2017.3.20160538

**Published:** 2017-07

**Authors:** Tonguc D. Berkol, Yasin H. Balcioglu, Simge S. Kirlioglu, Habib Erensoy, Meltem Vural

**Affiliations:** *From the Department of Psychiatry, Bakırkoy Research and Training Hospital for Psychiatry, Neurology and Neurosurgery (Berkol, Balcioglu, Kirlioglu), from the Department of Psychiatry (Erensoy), Neuropsychiatry Hospital, Uskudar University, and from the Department of Physical Medicine and Rehabilitation (Vural), Physical Medicine and Rehabilitation Hospital, Istanbul, Turkey*

## Abstract

**Objective:**

To assess the relationships between the dissociative features of FMS and the pain, psychological status, and functional status.

**Methods:**

Twenty-seven women with fibromyalgia syndrome (FMS) and 24 controls from the Istanbul Physical Medicine and Rehabilitation Hospital (2013-2015) were included in this cross-sectional study. The Diagnostic and Statistical Manual of Mental Disorders Structured Clinical Interview for Axis I Disorders was used to evaluate the participants. A visual analogous scale (VAS), Fibromyalgia Impact Questionnaire (FIQ), Beck Depression Inventory (BDI), and Beck Anxiety Inventory (BAI) were used to assess the levels of pain, quality of sleep and functional and psychological statuses. The Dissociative Experiences Scale (DES) was used to evaluate the dissociative features.

**Results:**

The BDI, BAI and DES scores were statistically significantly higher in the cases of FMS. There were remarkable associations between all but 2 of the DES and FIQ scores, while positive correlations were found between the DES and, VAS pain and sleep quality scores. The prevalences of current and lifelong dysthymia, and major depressive disorder; not otherwise specified, common anxiety and somatoform disorders were higher in the cases of FMS.

**Conclusion:**

Pain, physical function and emotional status appear to be associated with dissociative features in FMS. Further studies are required to define these relationships and improve treatment.

Fibromyalgia syndrome (FMS) is a disorder characterized by chronic and widespread musculoskeletal pain and other concomitant symptoms.^1^ It is reported to be more common in women than men.^2^ According to the 1990 American College of Rheumatology (ACR) criteria, in addition to widespread pain in 4 quadrants of the body for at least 3 months, the pain sensitivity is determined by a finger pressure of 4 kilograms in at least 11 of 18 points.^3^ Pain, fatigue, and non-relaxing sleep problems constitute the main FMS symptomatology, but it is accompanied by other symptoms, such as headaches, paresthesia, and an irritable colon or bladder.^4^,^5^ These symptoms have gained importance according to the recently published ACR diagnosis criteria.^6^ Fibromyalgia syndrome is a medically unexplained condition, with no biological tests for establishing its diagnosis. In the etiopathogenesis, several entities have been mentioned, including central and autonomous nervous system dysfunction, and various neurotransmitter imbalances, hormones, and psychiatric features.^7^ It is known that FMS, which is one of the most common reasons for widespread chronic pain, can be accompanied by different psychiatric disorders. Fibromyalgia syndrome substantially reduces the quality of life, thereby increasing the diagnosis and treatment costs.^8^ The prevalence of emotional distress is high, and mood and/or anxiety disorders frequently accompany FMS. However, many authors have emphasized that these diagnoses may occur prior to (or consecutively with) an FMS diagnosis; therefore, joint and advanced treatment strategies must be developed.^9^-^11^ Dissociation is defined as the breakage of generally integrated functions of consciousness, memory, identity, or environmental perception. Impairment of these integrative and connective functions in the mind may vary, ranging from normal values to pathological situations. Fibromyalgia syndrome has been associated with childhood traumas, more frequently than in other chronic pain groups, and it has been stated that traumas may constitute an independent risk for the development of FMS.^12^-^15^ In a limited number of studies, it has been reported that dissociative symptoms are frequently seen in FMS.^16^,^17^ Considering the frequent incidence of childhood traumas in these patients, and the fact that FMS is accompanied by various different psychiatric disorders, it may be assumed that the rates of dissociation will be high in FMS. In addition, it may be related to the neurodevelopmental and experience-related factors of early childhood, such as dissociative experiences attachment properties. The aim of this study was to examine the effects of dissociative features on the pain, sleep quality, symptoms, and emotional and functional properties of FMS patients, and to compare the results with healthy control subjects.

## Methods

Twenty-seven female patients with FMS diagnoses according to 1990 the ACR criteria, between 20-45 years of age, who applied to the Istanbul Physical Medicine and Rehabilitation Hospital, Physical Medicine and Rehabilitation outpatient clinic between 2013 and 2015, were included in this study. In addition, 24 healthy control group subjects, age and gender-matched to the patients, with no psychiatric disorder histories and no pain complaints, volunteered to participate. Regional ethical committee approval was obtained for this study. The inclusion criteria were an FMS diagnosis according to the 1990 ACR criteria in the patients, and an education level of elementary school or above for all of the participants. The exclusion criteria were pregnancy, ongoing suicidal ideation, psychotic disorder, neurological disease, the use of neurological and/or psychiatric drugs, severe systemic disease, and abnormalities in the routine analyses. The demographical features of the patients, symptoms accompanying FMS, and number of sensitive points were determined, and a visual analog scale (VAS) was used to assess the pain and sleep quality. The Fibromyalgia Impact Questionnaire (FIQ) was used to evaluate the health-related physical functions, and the Diagnostic and Statistical Manual of Mental Disorders (DSM)-IV Structured Clinical Interview for Axis I Disorders (SCID-I) was used to determine whether or not a patient had an axis I psychiatric disease. The Beck Depression Scale (BDS) and Beck Anxiety Scale (BAS) were applied in order to determine the anxiety and depression levels of the patients. The Dissociative Experiences Scale (DES) was used to assess the dissociative experiences and disorders. The psychiatric diagnosis distribution, dissociative features, and depression and anxiety levels of the FMS patients were determined, and compared to the control group.

In this study, the statistical analyses were conducted using the Number Cruncher Statistical System (NCSS) 2007 statistical software program (Utah, USA). In addition to the descriptive statistical methods (average, standard deviation) used in the assessment of the data, an independent t test was used for the comparison of the dual groups, the chi-squared test was used to compare the qualitative data, and the pearson correlation test was used to determine the interrelationships of the variables. The results were assessed at a significance level of *p*<0.05.

## Results

**Figure 1:**
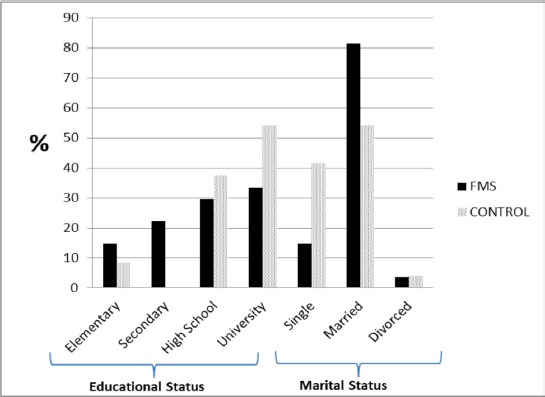
The anthropometric data and demographical features of the FMS and control groups. FMS - fibromyalgia syndrome

**Figure 2:**
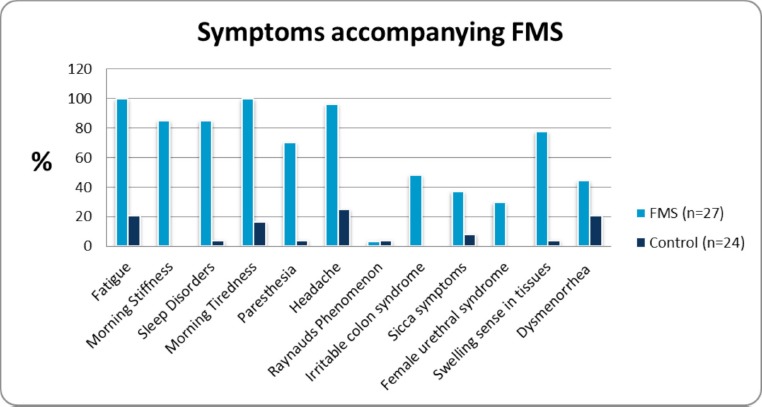
Symptoms accompanying FMS. *p<0,05, FMS - fibromyalgia syndrome

The anthropometric data and demographical features of the FMS and control groups are shown in **Table 1** and **Figure 1**. The average of the sensitive points detected by palpation in the FMS group was found to be statistically significantly higher than in the control group (*p*<0.001). In addition, the VAS pain and sleep quality averages of the FMS group were found to be statistically significantly higher than in the control group (*p*<0.001) (**Table 1**). The incidences of fatigue, morning stiffness, sleep disorders, morning tiredness, paresthesia, irritable colon syndrome (ICS), sicca symptoms, female urethral syndrome, swelling sense in the tissues, and headaches in the FMS group were found to be statistically significantly higher than in the control group (*p*<0.001). Statistically insignificant differences were observed between the FMS and control groups in terms of Raynaud’s Phenomenon (*p*>0.05) and dysmenorrhea (*p*>0.05) (**Figure 2**).

**Table 1:** Anthropometric data, clinical findings and beck depression Scale, beck anxiety scale and dissociative experiences scale scores in FMS and control groups.

Variables	FMS (n=27)	Control (n=24)	*P*-value
	(Average±SD)		
Age (years)	34.59±5.08	33.21±7.88	0.455
Height (cm)	161.26±5.88	163.33±4.84	0.179
Bodyweight (kg)	64.04±7.78	60.54±9.34	0.151
No. Sensitive points by palpation	14.78±1.25	1.21±1.44	0.0001*
VAS pain	7.19±2.3	1.33±1.69	0.0001*
VAS sleep quality	6.04±2.39	1.33±2.32	0.0001*
BDS	19.63±8.55	7.38±6.84	0.0001*
BAS	24.19±12.95	7.63±6.91	0.0001*
DES	16.48±14.46	6.28±7.81	0.003*

* *p*<0.05, FMS - Fibromyalgia syndrome, VAS - Visual analogous scale, BDS - Beck depression scale, BAS - Beck anxiety scale, DES - Dissociative experiences scale, SD - Standard deviation

In the FMS patients, the FIQ physical functioning, well-being, missed days of work, job difficulty, pain, fatigue, resting, stiffness, anxiety, and depression parameters, and the total FIQ averages were found to be statistically significantly higher than in the control group (*p*<0.001). However, a statistically significant difference was not found between the missed days of work parameter averages of the FMS and control groups (*p*>0.05) (**Table 2**). The BDS *p*<0.001, BAS *p*<0.001, and DES *p*<0.05 averages of the FMS patients were found to be statistically significantly higher than in the control group (**Table 1**).

**Table 2:** Comparison of components of fibromyalgia syndrome impact questionnaire form in FMS and control groups.

FIQ	FMS	Control	*P*-value
	(Average±SD)		
Physical functioning	5.13±1.71	2.8±1.62	0.0001*
Well-being	6.98±2.07	2.2±2.11	0.0001*
Missed days of work	1±2.16	0.58±2.0	0.592
Job difficulty	5.7±2.5	1.75±1.59	0.001*
Pain	7.41±2.04	1.63±1.53	0.0001*
Fatigue	8.37±1.55	2.33±2.08	0.0001*
Resting	8.74±1.63	2.08±2.45	0.0001*
Stiffness	6.56±2.98	1.29±1.78	0.0001*
Anxiety	8.07±1.8	2.63±2.43	0.0001*
Depression	7.56±1.8	1.63±2.08	0.0001*
Total FIQ Score	70.72±13.89	19.28±12.96	0.0001*

* *p*<0.05, FIQ - Fibromyalgia syndrome impact questionnaire form, SD - Standard deviation, FMS - Fibromyalgia syndrome

**Figure 3:**
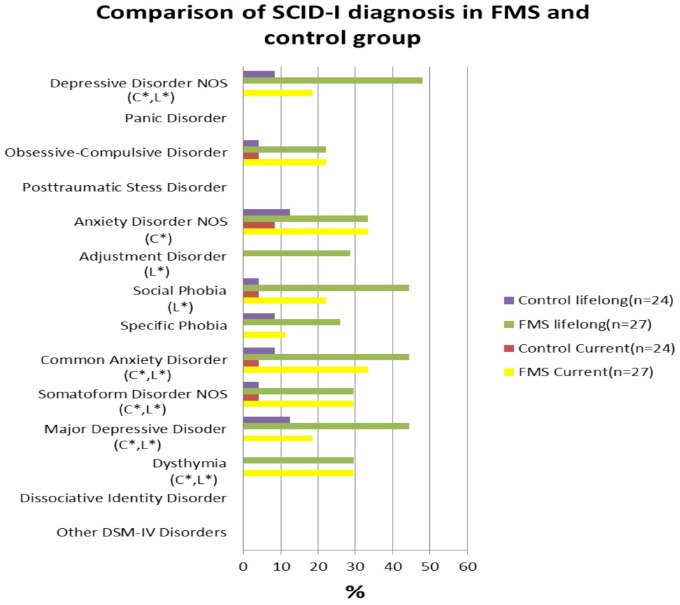
Comparison of SCID-I diagnoses in FMS and control groups. C - Statistical comparison of Current SCID-I diagnoses between FMS and control group, L - Statistical comparison of Lifelong SCID-I diagnoses between FMS and control group, NOS - Not otherwise specified, *p<0.05

In the FMS patients, with regard to the SCID-I diagnoses, the prevalences of both current and lifelong major depressive disorders, dysthymia, not otherwise specified (NOS) depressive disorder, common anxiety disorder, and somatoform disorder were statistically significantly higher than in the control group (*p*<0.05). The prevalences of NOS anxiety disorder in current SCID-I and adjustment disorder with social phobia in lifelong SCID-I were higher in the cases of FMS. However, current and lifelong SCID-I panic disorders, post-traumatic stress disorder, and dissociative identity disorder diagnoses were not observed in either group (**Figure 3**).

A positive and statistically significant relationship was determined between the DES and VAS pain (r=0.465, *p*<0.001) and sleep quality scores (r=0.408, *p*<0.001). Similarly, a positive and statistically significant relationship was found between the DES score and the number of palpation points (r=0.419, *p*<0.001).

Overall, a positive and statistically significant relationship was seen between the DES scores and the total FIQ scores (r=0.427, *p*<0.01) and FIQ parameters, other than the well-being (r=0.256, *p*>0.05) and missed days of work (r=0.035, *p*>0.05) parameters. There was also a positive and statistically significant relationship between the DES and the BDS (r=0.523, *p*<0.001) and BAS scores (r=0.656, *p*<0.001).

## Discussion

Physical and mental responses to stress may lead to various psychosomatic consequences. For example, FMS itself is recognized as a somatoform disorder. In the pathophysiology of FMS, factors like neuroendocrine, genetic, and psychosocial variables, environmental stressors, and autonomous and central nervous system abnormalities have been blamed. Among FMS patients, continuous or repetitive pain is often accompanied by emotional distress, as well as some psychiatric disorders. FMS has been associated with several psychological factors, including perfectionism, neuroticism, catastrophic thinking, and vigilance to pain.^19^

Somatoform dissociation represents an overmodulation of emotion, which is an intrapsychic process of traumatic stress, and leads to psychosomatic disorders such as FMS.^20^ In our study, the average DES score was found to be significantly higher than in the control subjects. In addition, Haviland et al^21^ reported a significant increase in the DES score in FMS patients suffering a major trauma. In another study, the average DES score of the FMS patients was found to be higher than in the rheumatoid arthritis (RA) patients, which was reported to be three times the general population average.^15^ Additionally, the average DES score of the FMS patients was found to be higher than in other rheumatologic diseases, and it was reported that this average was equal to 4 times the general population average.

A positive relationship was observed between the dissociation rates and medical symptom notification, and these symptoms were related to dissociation.^22^ In addition, a positive and statistically significant relationship was observed between the number of sensitive points, VAS pain and sleep quality, and DES averages in our study. Furthermore, a positive and statistically significant relationship was found between the DES and FIQ physical function, exercise, pain, fatigue, resting, malfunction, anxiety, and depression parameters and the total FIQ scores. In another study, the average DES score and total FIQ score were found to be higher in the FMS patients than in the control group subjects. However, in that study, the DES and FIQ correlation was not studied, but it was stated that post-traumatic stress disorder and physical activity may play roles in the development of FMS.^21^ In FMS patients, a relationship was seen between dissociation and pain, fatigue, and depression, while a significant relationship was not seen with sleep quality.^16^

The existence of a strong and direct correlation between trauma and dissociation has frequently been emphasized.^23^ The reason for this is that dissociation constitutes a basic adaptive response to trauma; therefore, dissociation ensures the reduction of an effect by the segmentation of traumatic experiences. At the same time, dissociation occurs in the form of disorders in the senses, movements, and other bodily functions. Anesthesia, analgesia, sensorial changes, and/or the loss of motor control constitute the basic symptoms of conversion disorder, and may be called somatoform dissociation.^24^

In our study, significant differences were seen between the FMS patients and control group subjects in terms of current and lifelong somatoform disorder diagnoses in the SCID-I. In one previous study, it was argued that post-traumatic stress disorder, dissociation, somatization, and affect dysregulation as a spectrum play roles in the adaptation to trauma.^25^ According to the authors, these phenomena are often seen together, and various combinations of them may develop in traumatized individuals overtime.

Depression and anxiety findings are seen in approximately 1/3 of FMS patients.^26^ In a study performed by Johnson et al,^27^ the severities of depression and anxiety were examined in healthy controls and FMS patients with chronic widespread musculoskeletal system pain, and found to be higher in the FMS patients. In addition, the current and lifelong major depression prevalences were determined to be between 20% and 80% in FMS patients when compared to healthy controls.^28^-^30^ Moreover, in one epidemiological study, FMS was described as the second most commonly observed general medical condition in relation to major depression.^31^

Considering the significance of the relationship between personality and depression in the general population, the significance of this relationship for FMS patients becomes obvious.^28^ In our study, in the FMS patients, the average BDS was 19.63±8.55, and the average BAS was 24.9±12.95. Busch et al^32^ reported an average BDS score of 20.6±3.1 in their FMS patients.In studies conducted on different disease groups, the relationships between depression and anxiety and dissociative disorders have been mentioned.^33^-^35^ We also determined a positive and significant relationship between the DES and the BDS and BAS scores in FMS patients. Moreover, anxious and depressive states have been found to be related to the symptomatology of FMS. Overall, the relationship between FMS and depressive disorders is more complicated than expected; therefore, more attention should be paid to the relationship between depression and anxiety and FMS.^36^

Our study did have several limitations. For example, the low numbers in both the patient and control groups, particularly the number of patients diagnosed with specific mental disorders, were the main restrictions of the current study. In addition, depersonalization and derealization were not included as dissociative disorder subtypes, due to their highly prevalent concurrence with other mental disorders as independent symptoms.

In conclusion, dissociative features seem to be related to pain, sleep disorders, functional situations, and depression in FMS patients. Describing the relationships between FMS and its concomitant symptoms, as well as emotional situations and dissociations, will be helpful in terms of both the FMS etiopathogenesis and treatment approaches.
